# The dual effects of leader bottom-line mentality on employee innovation behavior: the mediating role of willingness to take risks and the moderating role of work values

**DOI:** 10.3389/fpsyg.2025.1513006

**Published:** 2025-07-24

**Authors:** Shiwen Luo, Qi Fan, David Yoon Kin Tong

**Affiliations:** ^1^Zhejiang Financial College, Hangzhou, China; ^2^Zhejiang Shuren University, Hangzhou, China; ^3^International University of Malaya-Wales, Kuala Lumpur, Malaysia

**Keywords:** leader bottom-line mentality, willingness to take risks, work values, proactive innovation behavior, reactive innovation behavior

## Abstract

**Introduction:**

In the VUCA era, employee innovation behavior is critical to a enterprise success. In China’s high power distance and collectivist culture, employee innovation behavior is often influenced by leadership authority, resulting in distinct patterns of proactive and reactive innovation behavior that differentially predict innovation performance. Innovation is influenced not only by leadership type but also by leadership mindset. As a unidimensional approach, leader bottom-line mentality focuses on bottom-line profits while neglecting other important factors. However, its distinct predictive relationships with proactive versus reactive innovation behaviors remain insufficiently examined.

**Methods:**

This study employed a two-stage survey method in which a questionnaire survey was conducted with employees from 13 innovation-driven enterprises, and 351 valid responses were ultimately collected. Using SPSS and MPLUS software, the data were analysed through reliability and validity tests, confirmatory factor analysis, descriptive statistics, and linear regression to validate the proposed research hypotheses.

**Results:**

Leader bottom-line mentality is significantly negatively associated with proactive innovation behavior but positively associated with reactive innovation behavior. Moreover, willingness to take risks mediates the relationship between leader bottom-line mentality and employee innovation behavior. Additionally, intrinsic work values moderate the relationship between managers’ bottom-line mentality and risk-taking, which promotes proactive innovation behavior and reduces reactive innovation behavior. On the other hand, extrinsic work values positively moderate the relationship, enhancing reactive innovation behavior and reducing proactive innovation behavior.

**Discussion:**

As a unidimensional mentality, leader bottom-line mentality exhibits a dual predictive pattern with respect to employee innovation behavior: it positively predicts reactive innovation behavior while negatively predicting proactive innovation behavior. Notably, these predictive relationships are contingent upon work values: intrinsic work values attenuate the observed dual pattern, whereas extrinsic work values amplify these associations.

## 1 Introduction

In the VUCA (volatility, uncertainty, complexity, ambiguity) era, innovation is a key factor for the survival and development of enterprises (Li et al., 2022). Since employees are the core driving force of enterprise innovation, effectively motivating their innovation behavior is crucial to innovation performance (Newman et al., 2020).

In China’s high-power-distance and collectivist cultural context, employee innovation behavior tends to be oriented towards authority and prone to compliance (Ling and Zhihong, 2023). Moreover, enterprises often compulsorily implement innovation in unsuitable domains because of policy mandates, resulting in suboptimal innovation performance (Shao et al., 2020). A representative case involves manufacturing firms that implemented robotic automation systems under the national “Made in China 2025” initiative. Empirical evidence shows that an inadequate skilled workforce and inflexible operational management significantly constrained the expected productivity gains in these technology-driven projects (Chen, 2017). This phenomenon reflects the dualistic nature of innovation behavior in Chinese organizational contexts—proactive innovation (intrinsically motivated voluntary initiatives) versus reactive innovation (externally pressured involuntary compliance) (Yang et al., 2020; Ma et al., 2023) —with significantly divergent effects on innovation performance (Gao and Zhao, 2024).

Research has identified factors influencing these two types of innovation, emphasizing leadership styles and neglecting the underlying cognitive schemata of managers (Zhang et al., 2021). Particularly noteworthy is the unidimensional leader bottom-line mentality (LBLM), characterized by an exclusive focus on bottom-line outcomes (e.g., financial performance) (Greenbaum et al., 2012), which may influence employee innovation choices through dual pathways: it may either suppress autonomous innovation motivation by overemphasizing bottom-line targets, thereby inducing reactive innovation, or, alternatively, stimulate context-specific innovation vitality through clear goal orientation.

This dual-path mechanism of LBLM remains underexplored in the innovation management literature. Given the prevalence of performance-driven management practices in Chinese organizational contexts, investigating the differential effects of LBLM on these innovation types can not only expand the theoretical dimensions of innovation research but also offer practical insights for organizations to balance innovation autonomy and goal orientation in VUCA environments (Liu et al., 2022; Waseel et al., 2024).

The effect of leadership on employee innovation behavior is not merely a direct outcome but rather a complex evolutionary process (De Jong and Den Hartog, 2007). This process involves the transmission of psychological factors, including individual cognition, emotions, and motivation (Wu et al., 2022). Therefore, to explore the effect of LBLM on employee innovation behavior, it is essential to uncover the “black box” of its internal processes to fully reveal the underlying mechanisms through which LBLM affects employee innovation behavior.

The literature has explored aspects such as workplace spirituality (Ling and Zhihong, 2023), relative deprivation and psychological safety (Wan et al., 2021), perceived creativity expectations (Wang et al., 2025), and harmonious passion (Wu and Shen, 2024). However, the critical factor of willingness to take risks has not yet been addressed.

Since innovation is often accompanied by uncertainty and potential failure risks, employees are only likely to attempt new ideas and methods if they are willing to take risks (Dewett, 2004). Moreover, the loss aversion and status quo bias inherent in LBLM will inevitably diminish employee risk-taking propensity. Consequently, willingness to take risks may serve as a critical mediator through which LBLM affects employee innovation behavior.

According to social information processing theory, leaders serve as key social information sources that shape employee perceptions of the work environment through their attitudes, expectations, and behaviors, which in turn influence employee attitudes, motivations, and behaviors (Lerman, 2007). Research indicates that leaders with a strong bottom-line mentality consistently focus on ensuring the achievement of bottom-line goals and tend to avoid high-risk decisions (Ling and Zhihong, 2023).

When employees receive risk-averse signals from their leaders, they often reduce their own willingness to take risks (Saif et al., 2024; Shurygin et al., 2022). This phenomenon is naturally detrimental to proactive innovation behavior, which requires making risky decisions in highly uncertain contexts and bearing the potential for failure (Ma et al., 2023). In contrast, reactive innovation behavior is usually carried out as part of the tasks assigned by the organization, is associated with relatively lower risks (Gao and Zhao, 2024) and does not require employees to be willing to take risks. Therefore, willingness to take risks may serve as an important mediator of the relationship between LBLM and employee innovation behavior.

Leadership effectiveness depends not only on the leaders’ individual traits and behaviors but also, more crucially, on the interactions and collaborations between leaders and employees (Fiedler, 1964). Social information processing theory provides a valuable framework for understanding this dynamic process.

According to social information processing theory, employees interpret social cues in the organizational environment (such as leadership intentions) to form their perceptions and attitudes towards work, which in turn influence their behaviors. In this process, work values play a crucial moderating role (Carter et al., 2024). As a core component of individual cognition, work values reflect expectations regarding work outcomes (Abessolo et al., 2017; Gesthuizen et al., 2019) and are typically categorized into intrinsic and extrinsic dimensions (Taris and Feij, 2001).

Employees with intrinsic work values regard their jobs as pathways to self-actualization; they pursue autonomy, growth, creativity, and demonstrate a greater willingness to take risks (Aldjic and Farrell, 2022). They view innovation as a core professional responsibility, particularly emphasizing transformational and sustainability-oriented innovations. According to social information processing theory, when leaders’ behaviors and intentions are interpreted, such employees focus more on whether leaders support their self-actualization and innovation needs (Khan et al., 2022). Even if a leader’s bottom-line mentality which emphasizes risk aversion and short-term goals, conveys conservative signals, employees with strong intrinsic work values will still maintain their high levels of innovation motivation and willingness to take risks (Singh et al., 2021), thereby fostering proactive innovation behavior.

In contrast, employees with extrinsic work values place greater emphasis on external job rewards (e.g., compensation, promotion), viewing jobs primarily as a means to fulfil material needs, which consequently diminishes their intrinsic motivation for innovation (Taris and Feij, 2001). According to social information processing theory, when leaders’ behaviors and intentions are interpreted, these employees focus more on whether leaders can provide clear material incentives and career development opportunities. LBLM intensifies their risk-aversion tendency through its singular emphasis on bottom-line targets. Employees perceive that innovations exceeding these targets may squander resources and jeopardize personal gains (Fischer et al., 2019) and consequently opt for safer, LBLM-compliant reactive innovation approaches. Thus, work values moderate willingness to take risks, resulting in divergent effects of LBLM on employee innovation behavior.

In summary, drawing on social information processing theory within China’s organizational context, this study distinguishes proactive innovation behavior from reactive innovation behavior, investigates the differential effects of LBLM on employee innovation behavior through the mediator of willingness to take risks, and reveals the moderating role of work values in this process.

Accordingly, the theoretical contributions of this study are threefold. First, it reveals the divergent effects of LBLM on proactive and reactive innovation behaviors, providing a new perspective for understanding the complex influence of leadership on innovation. Second, by introducing willingness to take risks into the research model, this study opens the “black box” of the relationship between LBLM and employee innovation behavior, thereby offering a theoretical foundation for a deeper understanding of this complex relationship. Third, by incorporating work values as a moderator, this study highlights the significant differences in willingness to take risks among employees with different value orientations, thus providing insights to guide managers in adopting differentiated leadership strategies tailored to employees with varying values in practice.

## 2 Theoretical analysis and hypotheses

### 2.1 Leader bottom-line mentality and employee innovation behavior

In Western research contexts, employee innovation behavior is often viewed as an individual proactive and voluntary behavior (Parker and Collins, 2010). However, this mainstream innovation theory falls short in explaining the widespread phenomenon of “reactive” innovation behavior in the Chinese context. Specifically, in the context of “state-driven” innovation, various industries have witnessed a surge of innovation activities driven by policies and regulations, but these innovations often fail to achieve the expected performance outcomes.

As a result, scholars have classified employee innovation behavior in Chinese organizations into proactive innovation behavior and reactive innovation behavior (Yang et al., 2023). Proactive innovation behavior refers to employees’ voluntary engagement in innovation activities and willingness to confront potential challenges (Shalley et al., 2009). This type of innovation is characterized by spontaneity, proactivity, and risk-taking, often manifesting as “extrarole” actions that go beyond the scope of employees’ formal job responsibilities. Employees engage in such behavior driven by organizational commitment, curiosity and intrinsic motivation, often exceeding expected risk-taking levels (Yang et al., 2023; Luo et al., 2023).

In contrast, reactive innovation behavior refers to employee reluctance to comply with innovation demands due to external pressures or organizational mandates (Yang et al., 2023). This type of behavior is characterized by nonidentification, reactivity, and obligation and often manifests as employees passively completing innovation tasks under organizational demands or external environmental pressures (Wansu et al., 2019). Employees exhibiting such behavior lack intrinsic motivation and innovate primarily to meet expectations or avoid penalties rather than because of authentic interest (Abadi et al., 2023). Although such behavior may temporarily advance organizational innovation goals in the short term, the lack of initiative and intrinsic drive often results in unsustainable innovation outcomes and may undermine long-term innovation willingness and job satisfaction (Yang et al., 2023).

Drawing on social information processing theory, we argue that LBLM shapes employee perceptions and attitudes towards innovation behavior by conveying specific social information, which employees interpret as signals about organizational priorities, thereby influencing their choices regarding innovation behavior. By transmitting social information centered on “risk avoidance” and “profit maximization,” LBLM directs employees to focus more on achieving bottom-line goals, as they realize that attaining these goals is the optimal choice within their job scope (Babalola et al., 2022).

In this process, proactively taking risks to explore innovation behavior unrelated to bottom-line goals may be perceived as misusing organizational resources (Frese et al., 1997). Proactive innovation behavior typically involves extrarole behavior, requiring individuals to assume risks and invest additional resources (Yang et al., 2023). However, when employees receive strong bottom-line signals, they tend to avoid risky behaviors unrelated to these goals (Eissa et al., 2019), thereby reducing their engagement in spontaneous innovation.

This tendency not only constrains employee autonomy but also erodes their intrinsic motivation for innovation, thereby suppressing proactive innovation behavior. Conversely, employees may resort to reactive innovation behavior—fulfilling innovation tasks passively under external pressure to meet bottom-line goals. Despite lacking intrinsic motivation (Wansu et al., 2019), such behavior is perceived as legitimate because it aligns with the risk-averse logic institutionalized by LBLM. Therefore, this study proposes the following hypothesis:

*H_1_: Leader bottom-line mentality is negatively related to employee proactive innovation behavior.*

*H_2_: Leader bottom-line mentality is positively related to employee reactive innovation behavior.*

### 2.2 The mediating effect of willingness to take risks

Willingness to take risks refers to an individual’s intrinsic psychological motivation to accept high risks, potential losses, and possible negative outcomes to achieve favourable results for the organization, such as improving innovation performance or capturing market opportunities (Smith et al., 2016).

According to social information processing theory, leaders, as key sources of social information within an organization, convey clear messages to employees through their attitudes, expectations, and behaviors, thereby influencing employees’ willingness to take risks and their innovation behaviors (Lerman, 2007). Leaders with a strong bottom-line mentality often prioritize goal attainment and exhibit greater risk aversion. Moreover, to address issues other than the bottom-line goals, they tend to adopt conservative approaches, demonstrating limited willingness to take risks (Greenbaum et al., 2012).

When employees perceive their leaders’ singular focus on bottom-line goals and risk avoidance, they recognize that the organization discourages—and may even penalize—any behaviors deviating from these priorities. Consequently, employees are likely to allocate their primary resources towards achieving the bottom-line goals, which naturally diminishes their willingness to engage in risk-taking behaviors in other domains.

Furthermore, leaders with a bottom-line mentality typically adopt a zero-sum mindset. This cognitive orientation, as evidenced by Greenbaum et al. (2012), intensifies interpersonal competition among employees while simultaneously fostering job insecurity (Zhang et al., 2023). The resulting climate of heightened competition and insecurity, in turn, suppresses employees’ willingness to take risks, particularly in the context of innovation.

Willingness to take risks constitutes a critical antecedent of employee innovation behavior. On the one hand, risk aversion significantly constrains proactive innovative behavior, as this type of behavior inherently involves substantial uncertainty and unpredictable outcomes (Fan et al., 2022). The essence of proactive innovation requires employees to make risky decisions that fundamentally contradict the disposition of risk-averse individuals. Therefore, when exposed to LBLM, employees tend to avoid risky behaviors unrelated to bottom-line goals, consequently diverting resources away from proactive innovation behavior (Yang et al., 2023).

On the other hand, risk aversion may paradoxically foster reactive innovation behavior—predefined tasks with low risk and clear procedures (Luo et al., 2023). Under the influence of LBLM, employees are more inclined to complete innovation tasks directly tied to bottom-line goals while avoiding ancillary risks. Although this behavioral pattern reflects extrinsic rather than intrinsic motivation (Wansu et al., 2019), its congruence with the core principles of LBLM enhances both its organizational acceptability and employee executability. Thus, the risk-averse orientation of LBLM has a dual effect: it suppresses proactive innovation while facilitating reactive innovation. Therefore, this study proposes the following hypotheses:

*H_3_: Willingness to take risks plays a mediating role in the relationship between leader bottom-line mentality and employee proactive innovation behavior.*

*H_4_: Willingness to take risks plays a mediating role in the relationship between leader bottom-line mentality and employee reactive innovation behavior.*

### 2.3 The moderating effect of work values

Work values represent the operationalization of personal values in occupational contexts, embodying employees’ fundamental needs regarding their desired work characteristics and attributes (Sagie et al., 1996). As a core psychological construct, work values serve dual functions: they not only determine employees’ fundamental work motivations but also function as evaluative criteria for selecting and assessing work-related behaviors and situations. This dual nature profoundly influences individual work attitudes, cognitive judgements, and behavioral patterns (Cemalcilar et al., 2018).

Work values are typically categorized into intrinsic and extrinsic dimensions. Intrinsic work values refer to employees’ valuations of nonmaterial work characteristics that satisfy higher-order psychological needs, including job diversity, autonomy, and creative expression (Taris and Feij, 2001). In contrast, extrinsic work values pertain to the importance placed on material compensation and career advancement opportunities, such as financial remuneration, promotion prospects, and organizational status (Taris and Feij, 2001). This dichotomy reflects the fundamental distinction between self-actualization needs and instrumental rewards in work motivation theory.

Drawing on social information processing theory, employees systematically assess organizational signals (e.g., leaders’ expectations and reward systems) to determine value congruence with their personal orientations. This evaluation process leads to corresponding adjustments in work behaviors, cognitive frameworks, and attitudinal dispositions. Specifically, employees with strong intrinsic work values often interpret social cues and information within the organizational environment to develop a deep identification with their work, placing greater emphasis on personal development opportunities, autonomy, responsibility, and a sense of achievement (Van den Broeck et al., 2021).

These employees demonstrate a greater propensity for proactive risk-taking behaviors, even when organizational leaders emphasize bottom-line goal attainment or institute penalties for extrarole initiatives. According to social information processing theory, they perceive innovation and risk-taking as crucial pathways to achieving self-worth and personal growth (Salancik and Pfeffer, 1978). Empirical evidence confirms that higher levels intrinsic work values correlate significantly with: (a) an increased organizational change orientation, (b) increased work responsibility, and (c) a greater risk-taking propensity (Lin et al., 2015; Gesthuizen et al., 2019). For example, employees with strong intrinsic work values may prefer roles that allow them to make independent decisions about their work content, despite potential compromises in job security.

In contrast, employees with strong extrinsic work values are concerned primarily with tangible work rewards (Gagné et al., 2022). Through systematic processing of organizational signals (e.g., leadership priorities, incentive structures), they rationally conclude that compliance with bottom-line goals represents the optimal strategy for reward maximization. Conversely, they perceive risk-taking behaviors and resource allocation beyond prescribed goals as suboptimal choices that jeopardize potential gains. For these employees, the calculus of concrete benefits consistently outweighs organizational change motivations, resulting in systematically diminished risk propensity. This pattern substantiates the critical moderating role of work values in the relationship between LBLM and willingness to take risks, with the intrinsic–extrinsic dichotomy exerting differential effects on both risk tolerance and innovation propensity.

*H_5_: Intrinsic work values moderate the relationship between leader bottom-line mentality and willingness to take risks, weakening its negative effect.*

*H_6_: Extrinsic work values moderate the relationship between leader bottom-line mentality and willingness to take risks, strengthening its negative effect.*

Building upon the above analysis and proposed hypotheses, this study establishes a mediated moderation model wherein LBLM indirectly affects employee innovation behavior (encompassing both proactive and reactive forms) through the pathway of willingness to take risks, with work values (intrinsic/extrinsic) serving as critical moderators.

Specifically, a high level of intrinsic work values can moderate the negative effect of LBLM on willingness to take risks, thereby enhancing proactive innovation behavior while weakening reactive innovation behavior. Conversely, a high level of extrinsic work values can strengthen the negative effect of LBLM on willingness to take risks, thereby diminishing proactive innovation behavior while promoting reactive innovation behavior. Therefore, this study proposes the following integrated hypotheses:

*H_7_: Intrinsic work values moderate the mediating role of willingness to take risks in the relationship between leader bottom-line mentality and employee innovation behavior (both proactive and reactive).*

*H_8_: Extrinsic work values moderate the mediating role of willingness to take risks in the relationship between leader bottom-line mentality and employee innovation behavior (both proactive and reactive)*.

**Figure 1:**
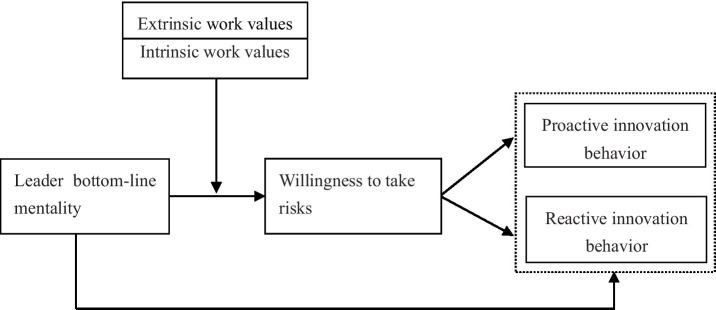
Theoretical model.

Based on the above discussion, the theoretical research model of this article is derived, as shown in Figure 1.

## 3 Method

### 3.1 Research process and samples

Considering the availability and authenticity of the data, this study adopted the convenience sampling method to conduct an onsite questionnaire survey among employees of 13 companies engaged in innovation activities between June and September 2023, covering high-tech services, electronic information technology, biotechnology and pharmaceuticals, and telecommunications. Approval for the study was obtained from the Ethics Committee of Zhejiang Financial College. Prior to completing the questionnaires, all the participants signed informed consent forms and were informed that the data would be used solely for academic purposes prior to completing the questionnaires.

To ensure data accuracy, this study followed the approach of Hussain et al. (2025) by implementing strict quality control measures throughout the scale design, questionnaire distribution, and data collection processes. In collaboration with a market research company, the questionnaires were distributed and collected on site. Additionally, to reduce the effect of common method bias and improve the reliability of the research results, this study employed a two-stage survey approach in which distinct types of data were collected in each stage. By temporally separating the data collection and employing anonymous coding, the correlation between datasets was minimized, thereby effectively controlling for common method bias.

The first phase (June 2023) collected data on the independent variable (LBLM), the mediator (willingness to take risks), the moderator (work values), and demographic characteristics. The second phase (August 2023) assessed the dependent variable (employee innovation behavior). Participant matching was ensured by implementing a two-month interval and an anonymous coding system (where employees entered their company abbreviation plus the last four digits of their employee ID) to maintain confidentiality while permitting data linkage. All the data were anonymized for research validation purposes. The original questionnaires were stored on password-protected servers with automatic encryption and are scheduled for secure destruction in compliance with institutional protocols.

In the first phase, 460 questionnaires were distributed. After excluding responses with invalid patterns (e.g., all responses were identical) or missing data, 412 valid questionnaires were retained, resulting in a usable response rate of 89.57%. The second phase was conducted 2 months later and primarily assessed the dependent variable (employee innovation behavior). Using the anonymous identifiers from the first phase, we matched second-phase responses with first-phase data while preserving anonymity to mitigate social desirability bias and privacy concerns. After excluding cases of attrition and invalid responses (due to response patterns or missing data), 351 valid questionnaires were obtained, yielding a final matched response rate of 85.19%.

The sample attrition in the second phase was due to the following reasons: (1) employee turnover or job relocation, (2) noncompletion resulting from time constraints or diminished survey engagement, and (3) selective attrition among employees with greater work pressure or lower organizational commitment, who may have been less willing to participate in additional tasks. Nevertheless, when the demographic characteristics (e.g., age, gender, tenure) of the samples were compared between Phase 1 and Phase 2, no significant differences were found, indicating that the sample attrition did not substantially compromise the sample’s representativeness (see Table 1).

**Table 1:** Sample distribution.

Variables	Details	Frequency	Percent
Gender	Male	233	66.38%
	Female	118	33.62%
Age	18–25	39	11.11%
	26–35	163	46.44%
	36–45	117	33.33%
	≥45	32	9.12%
Education	Master	86	24.50%
	Bachelor	178	50.71%
	≤College degree	87	24.79%
Work tenure	≥3 years	139	39.60%
	1-3 years	167	47.58%
	< 1 year	45	12.82%

### 3.2 Measurements

#### 3.2.1 Leader bottom-line mentality (LBLM)

This article uses a scale developed by Greenbaum et al. (2012) to measure LBLM; these items were originally created to reflect respondents’ assessment of LBLM. The respondents indicated how strongly they agreed that their leader (a) “is solely concerned with meeting the bottom line”; (b) “only cares about the business”; (c) “treats the bottom line as more important than anything else”; and (d) “cares more about profits than employee well-being.” The Cronbach’s *α* was 0.813.

#### 3.2.2 Willingness to take risks

The willingness to take risks was measured with a scale developed by Dewett (2004). The scale consists of 8 items: (a) “When I think of a good way to improve the way I accomplish my work, I will risk potential failure to try it out”; (b) “I will take a risk and try something new if I have an idea that might improve my work, regardless of how I might be evaluated”; (c) “I will take informed risks at work in order to get the best results, even though my efforts might fail”; (d) “I am willing to go out on a limb at work and risk failure when I have a good idea that could help me become more successful”; (e) “I do not think twice about taking calculated risks in my job if I think they will make me more productive, regardless of whether or not my efforts will be successful”; (f) “Even if failure is a possibility, I will take informed risks on the job if I think they will help me reach my goals”; (g) “When I think of a way to increase the quality of my work, I will take a risk and pursue the idea even though it might not pan out”; and (h) “In an effort to improve my performance, I am willing to take calculated risks with my work, even if they may not prove successful.” The Cronbach’s *α* was 0.871.

#### 3.2.3 Work values

The measurement of intrinsic and extrinsic work values was based on the research of Chang et al. (2008). The intrinsic work values scale included the following 7 items: (a) “opportunities to learn new technology and knowledge”; (b) “the R&D project itself”; (c) “autonomy in performing tasks”; (d) “possibilities for expressing and realizing my full capability”; (e) “opportunities to achieve something valuable”; (f) “opportunities to participate in decision-making”; and (g) “participation in professional/academic conferences or seminars.” The Cronbach’s *α* was 0.859. Extrinsic work values were measured by the following 5 items: (a) “salary”; (b) “fringe benefits (e.g., pension, insurance, paid leave)”; (c) “social reputation of my job”; (d) “status at the laboratory or the company”; and (e) “satisfaction of my family regarding my job.” The Cronbach’s α was 0.865.

#### 3.2.4 Employee innovation behavior

Proactive innovation behavior was measured mainly following Frese et al. (1997) via 7 items: (a) “I actively attack problems”; (b)” Whenever something goes wrong, I search for a solution immediately”; (c) “Whenever there is a chance to get actively involved, I take it”; (d) “I take initiative immediately even when others do not”; (e) “I use opportunities quickly in order to attain my goals”; (f) “Usually, I do more than I am asked to do”; and (g) “I am particularly good at realizing ideas.” The Cronbach’s α was 0.846; The measurement of reactive innovation behavior mainly draws on the research results of Zhao et al. (2015), with a total of 5 items, including (a)"Innovation is just about completing established tasks”; (b) “Utilizing loopholes in innovation rules and perfunctory innovation”; (c) “To achieve and recognize innovative achievements, one has to change their creative ideas to comply with social norms and industry paradigms”; and (d) “Innovation should cater to the preferences of leaders and execute their innovative instructions like a machine”; (e) “Engaging in innovative work under high pressure and strict reward and punishment systems.” The Cronbach’s α was 0.834.

#### 3.2.5 Control variables

Based on the theoretical foundations and previous studies, this study selected employee gender, age, education, and work tenure as control variables. Specifically: (1) gender may influence employees’ work attitudes and behavioral patterns (Eagly and Wood, 2012), and thus needs to be controlled for; (2) work tenure reflects employees’ accumulated experience and career development stages (Ng and Feldman, 2010), which may systematically affect the research variables; and (3) Education, as a key indicator of human capital (Becker, 1964), may influence employees’ work cognition and behavioral decision-making. By controlling for these variables, we can more accurately estimate the net effect of the independent variable on the dependent variable, thereby enhancing the internal validity and theoretical explanatory power of the research findings.

The above scales were all scored on a 5-point Likert scale (1 not important, 5 very important).

## 4 Results

### 4.1 Confirmatory factor analysis

This study used Mplus 7.0 to conduct confirmatory factor analysis. The fitted values of each model are shown in Table 2. According to Table 2, compared with other competitive models, the six-factor model has the best fit (χ^2^/df = 2.017, RMSEA = 0.058, NNFI = 0.931, CFI = 0.928, IFI = 0.910). Moreover, all the fit indicators of the model have reached the standards recognized by the academic community. Therefore, the variables in this study show discriminant validity, which can be used for the next step of testing the correlation between variables.

**Table 2:** The results of confirmatory factor analysis.

Models	*χ*^2^/ *df*	RMSEA	NNFI	CFI	IFI
LBLM; WTR; IWV; EWV; PIB; RIB	2.017	0.058	0.931	0.928	0.910
LBLM; WTR; IWV; EWV; PIB + RIB	2.311	0.134	0.902	0.911	0.902
LBLM; WTR; IWV; EWV + PIB + RIB	2.572	0.199	0.853	0.849	0.848
LBLM; WTR; IWV + EWV + PIB + RIB	3.176	0.204	0.807	0.812	0.810
LBLM; WTR + IWV + EWV + PIB + RIB	3.520	0.238	0.733	0.721	0.732
LBLM+WTR + IWV + EWV + PIB + RIB	4.532	0.273	0.474	0.469	0.470

LBLM, leader bottom-line mentality; WTR, willingness to take risks; IWV, intrinsic work values; EWV, extrinsic work values; PIB, proactive innovation behavior, RIB, reactive innovation behavior.

Although the data were collected at two time points, considering that LBLM, willingness to take risks, work values (intrinsic and extrinsic work values), and employee innovation behavior (proactive and reactive innovation behaviors) were all reported by the employees themselves, there may still be a risk of common method bias. Therefore, a Harman single-factor test was conducted. The results revealed that the variance explained by the first common factor was 23.63%, which was below the critical value of 40%, indicating that the problem of common method bias was not serious.

To further enhance the rigor and credibility of the research results, a common method factor was added to the six-factor model to further test for common method bias. Unfortunately, the seven-factor model could not be fitted with Mplus 7.0 software. Thus, the factor structure with common method factors did not significantly change in terms of the fitting degree, indicating that there is no serious problem of common method bias in this study.

### 4.2 Descriptive statistics

The means and correlation coefficients for the main variables in this study are shown in Table 3. The results of independent samples t-tests revealed that: LBLM has a significant negative correlation with proactive innovation behavior (*r* = −0.195, *t* = −4.331, *p* < 0.01), a positive correlation with reactive innovation behavior (*r* = 0.249, *t* = 5.613, *p* < 0.01), and a negative correlation with willingness to take risks (*r* = −0.438, *t* = −9.884, *p* < 0.01). In addition, willingness to take risks is positively correlated with proactive innovation behavior (*r* = 0.383, *t* = 8.540, *p* < 0.01), and negatively correlated with reactive innovation behavior (*r* = −0.137, *t* = −2.991, *p* < 0.05). The statistical analyses followed established significance thresholds and effect size criteria (Farooq et al., 2022; Saif et al., 2025). All correlations maintained statistical significance after multiple comparison adjustments, with variance inflation factors indicating no multicollinearity concerns.

**Table 3:** The results of descriptive statistics.

Variables	M	GEND	EDU	WT	LBLM	WTR	IWV	EWV	PIB	RIB
GEND	0.662	1.000								
EDU	2.405	0.069	1.000							
WT	2.269	0.077	0.054	1.000						
LBLM	3.986	0.104^*^	0.066	−0.017	1.000					
WTR	2.878	−0.094	0.061	0.033	−0.438^**^	1.000				
IWV	3.413	−0.078	0.084	0.019	0.218^**^	0.088	1.000			
EWV	3.641	0.044	−0.065	0.026	0.126^*^	0.144^*^	−0.029	1.000		
PIB	3.756	0.009	0.073	−0.014	−0.195^**^	0.383^**^	0.308^**^	−0.352^**^	1.000	
RIB	2.942	0.072	0.023	0.055	0.249^**^	−0.137^*^	−0.141^*^	0.268^**^	0.062	1.000

*P < 0.05; **P < 0.01(two-tailed tests); GEND, gender diversity; EDU, education; WT, work tenure.

### 4.3 Hypothesis testing

This study employs linear regression analysis to test the hypotheses, as this method effectively quantifies causal relationships between variables and evaluates the goodness-of-fit of predictive models (see Alam et al., 2023; Saif et al., 2024). The results are shown in Table 4.

**Table 4:** The results of linear regression analysis.

Variables	PIB		RIB		WTR				
	M_1_	M_2_	M_3_	M_4_	M_5_	M_6_	M_7_	M_8_	M_9_
GEND	0.047 (0.603)	−0.006 (−0.191)	−0.027 (−0.426)	0.033 (0.512)	−0.038 (−0.722)	−0.004 (−0.107)	0.017 (0.383)	−0.024 (−0.414)	0.046 (0.600)
EDU	0.042 (0.352)	0.034 (0.409)	0.025 (0.292)	−0.002 (−0.100)	0.036 (0.631)	0.021 (0.457)	0.050 (1.024)	0.063 (1.243)	0.042 (0.658)
WT	0.059 (1.121)	0.006 (0.205)	−0.019 (−0.329)	0.035 (0.830)	0.013 (0.479)	0.041 (0.856)	0.044 (0.885)	0.039 (0.692)	0.038 (0.701)
LBLM	−0.206^**^ (−3.699)	−0.286^**^ (−4.291)	0.477^**^ (8.572)	0.322^**^ (6.440)	−0.385^**^ (−6.932)	−0.243^**^ (−4.017)	−0.352^**^ (−6.238)	−0.271^**^ (−4.130)	−0.306^**^ (−5.236)
WTR		0.411^**^ (7.426)		−0.293^**^ (−4.873)					
IWV						0.162^**^ (2.865)	0.144^*^ (2.542)		
EWV								−0.218^**^ (−3.884)	−0.148^**^ (−2.677)
LBLM* IWV							−0.189^**^ (−3.171)		
LBLM* EWV									0.136^*^ (2.328)
*R* ^2^	0.193	0.622	0.727	0.074	0.290	0.288	0.577	0.593	0.681
△*R*^2^	0.190	0.597	0.701	0.067	0.274	0.249	0.508	0.529	0.647
*F*	4.996	6.322	7.870	6.943	5.442	10.255	8.734	8.044	5.588

The equations are estimated using OLS, and the test statistics are based on the two-sided t-stat; *P < 0.05; **P < 0.01.

In Table 4, as shown in M_1_, after controlling for relevant variables (gender, education, and work tenure), LBLM has a significant negative association with employee proactive innovation behavior (*β* = −0.206, *p* < 0.01). As shown in M_3_, LBLM has a significant positive association with employee reactive innovation behavior (*β* = 0.477, *p* < 0.01). Therefore, Hypothesis 1 and Hypothesis 2 are supported.

According to M_5_, LBLM is significantly negatively associated with willingness to take risks (*β* = −0.385, *p* < 0.01); according to M_2_, willingness to take risks is significantly positively associated with employee proactive innovation behavior (*β* = 0.411, *p* < 0.01); and compared with M_1_, the association between LBLM and proactive innovation behavior remains statistically significant (*β* = −0.286, *p* < 0.01), indicating that willingness to take risks plays a mediating role in the relationship between LBLM and proactive innovation behavior, thus supporting Hypothesis 3. Moreover, according to M_4_, it can be inferred that willingness to take risks is negatively associated with reactive innovation behavior (*β* = −0.293, *p* < 0.01), and compared with that of M_3_, the association between LBLM and reactive innovation behavior remains statistically significant (*β* = 0.322, *p* < 0.01), indicating that willingness to take risks also plays a mediating role in the relationship between LBLM and reactive innovation behavior, thus supporting Hypothesis 4.

To further verify the mediating role of willingness to take risks, the data were analysed via the process method in SPSS and sampled 5,000 times to examine their significance. The results indicated that after controlling for relevant variables, the indirect effect of LBLM on proactive innovation behavior through willingness to take risks was −0.108, with a 95% CI of [0.022, 0.074], excluding 0; the indirect effect of LBLM on reactive innovation behavior through willingness to take risks was 0.069, with a 95% CI of [0.019, 0.108], excluding 0. Therefore, Hypotheses 2 and 3 are once again supported.

This study further tested Hypotheses 5 and 6 with the moderating effect test method. To avoid multicollinearity, LBLM and work values (internal and extrinsic work values) were centralized. The results are shown in Table 4. Table 4 shows that after controlling for relevant variables, the interaction term (LBLM* internal work values) has a significant negative association with willingness to take risks (*β* = −0.189, *p* < 0.01). According to M_9_, there was a significant positive association between the interaction item (LBLM* extrinsic work values) and willingness to take risks (*β* = 0.136, *p* < 0.05).

**Figure 2:**
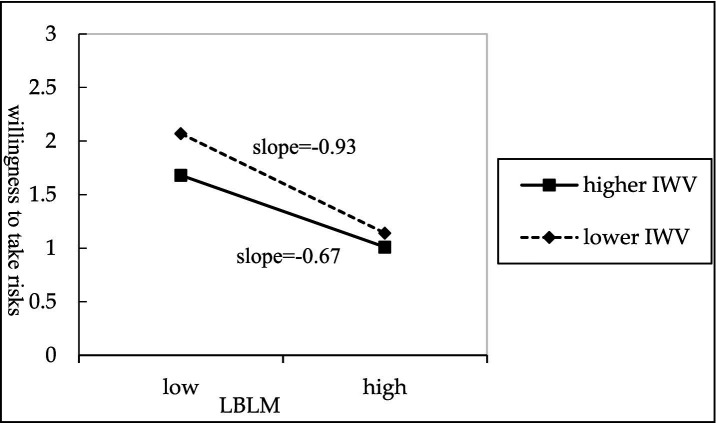
The moderating effect of intrinsic work value.

**Figure 3:**
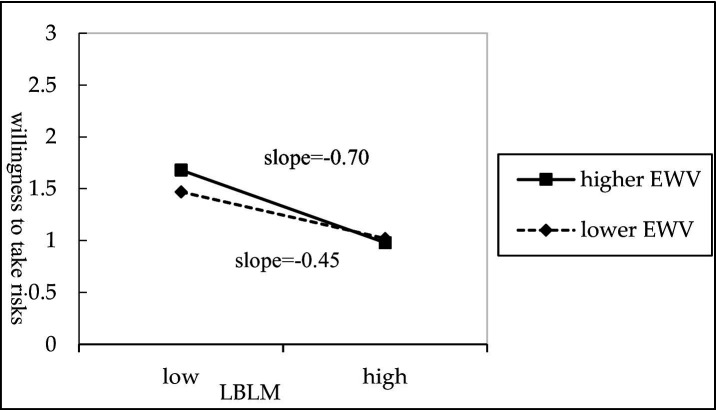
The moderating effect of extrinsic work value.

To further clarify the direction and magnitude of the moderating effect, this study used the mean plus or minus one standard deviation as the benchmark to separate high and low levels of employee work values (intrinsic and extrinsic work values). The simple slope test results are shown in Figures 2, 3. Figure 2 shows that the negative association between LBLM and willingness to take risks is stronger when employees exhibit lower levels of intrinsic work values, whereas this association weakens among those with higher intrinsic work values. Therefore, Hypothesis 5 is supported. Furthermore, as shown in Figure 3, the negative association between LBLM and willingness to take risks is more pronounced when employees demonstrate higher levels of extrinsic work values, whereas this association is lower among employees with lower extrinsic work values. Therefore, Hypothesis 6 is supported.

The moderated mediation effect was tested via the path analysis method proposed by Edwards and Lambert (2007), and the results are shown in Table 5. The indirect effect of LBLM on proactive innovation behavior was not significant under high intrinsic work values (*β* = −0.021, 95% CI = [−0.162, 0.027]) but was significant under low intrinsic work values (*β* = 0.068, 95% CI = [0.022, 0.046]). The indirect effect of LBLM on reactive innovation behavior was also not significant under high intrinsic work values (*β* = 0.009, 95% CI = [−0.017, 0.069]) but was significant under low intrinsic work values (*β* = 0.059, 95% CI = [0.014, 0.045]). Hence, Hypothesis 7 was supported.

**Table 5:** The results of moderated path analysis.

Variables	LBLM—WTR—PIB		LBLM—WTR—RIB	
	Indirect effect	95%CI(BCB)	Indirect effect	95%CI(BCB)
Higher IWV (+1 SD)	−0.021	[−0.162, 0.027]	0.009	[−0.017, 0.069]
Lower IWV (−1 SD)	0.068	[0.022, 0.046]	0.059	[0.014, 0.045]
Diff	0.093	[0.015, 0.253]	0.128	[0.008, 0.073]
Higher EWV (+1 SD)	−0.071	[−0.032, −0.016]	−0.255	[−0.043, −0.003]
Lower EWV (−1 SD)	0.011	[−0.012, 0.029]	0.013	[−0.025, 0.054]
Diff	0.048	[−0.026, −0.012]	0.083	[−0.043, −0.003]

The indirect effect of LBLM on proactive innovation behavior was significant under high extrinsic work values (*β* = −0.071, 95% CI = [−0.032, −0.016]) but not significant under low extrinsic work values (*β* = 0.011, 95% CI = [−0.012, 0.029]). The indirect effect of LBLM on reactive innovation behavior was also significant under high extrinsic work values (*β* = −0.255, 95% CI = [−0.043, −0.003]) but not significant under low extrinsic work values (*β* = 0.013, 95% CI = [−0.025, 0.054]). Thus, Hypothesis 8 was supported.

## 5 Conclusion and insights

### 5.1 Conclusion

First, our model shows that LBLM has differential effects on innovation types. Specifically, LBLM negatively predicts proactive innovation behavior while positively predicting reactive innovation behavior. This directional pattern suggests that LBLM creates an organizational climate that systematically favors compliant, reactive innovation over self-initiated proactive efforts. As key social information transmitters, leaders communicate priority expectations through their attitudes and behaviors. When exposed to LBLM, employees cognitively reconstruct their behavioral strategies by (a) prioritizing bottom-line goal attainment (e.g., profit targets, financial metrics), (b) demonstrating compliance with prescribed innovation tasks, and (c) systematically avoiding the uncertainties inherent in proactive innovation that might jeopardize core performance metrics. This risk-averse calculus emerges from anticipated organizational sanctions against goal-discrepant behaviors.

Second, willingness to take risks mediates the relationship between LBLM and both forms of innovation behavior. Under the influence of LBLM, employees develop cognitive appraisals that extra role risk-taking may incur organizational sanctions. As a result, they become more conservative, and their willingness to take risks decreases. Given that risk-taking willingness is a key factor driving innovation activities (Dewett, 2006), when their willingness to take risks diminishes, they are more inclined to perform tasks assigned by the organization and thus engage in low-risk, reactive innovation rather than actively taking risks to pursue high-risk and more uncertain innovation activities.

Third, work value orientation (intrinsic vs. extrinsic) significantly moderates the predictive relationship between LBLM and employee innovation behavior through willingness to take risks. Employees with predominant intrinsic work values prioritize self-determination, professional growth, and creative expression (Twenge, 2010). This value configuration corresponds with a weaker predictive relationship between LBLM and willingness to take risks, aligning with (a) enhanced engagement in proactive innovation and (b) reduced reliance on reactive innovation. Conversely, strong extrinsic work values orient employees towards economic security and material outcomes, leading to more conservative behavior, which amplifies the negative predictive association between LBLM and willingness to take risks. This situation promotes reactive innovation while suppressing proactive innovation.

### 5.2 Theoretical contributions

First, this study makes three key theoretical contributions to the LBLM literature. First, it identifies a dual-path predictive pattern between LBLM and innovation behavior, thus challenging the conventional conceptualization that equates employee innovation solely with proactive forms (Parker and Collins, 2010; Kwon and Kim, 2020). By introducing the proactive reactive innovation dichotomy within China’s unique organizational context, we provide a more nuanced understanding that addresses calls for exploring LBLM’s predictive relationships beyond traditional outcome variables (Xiaojun et al., 2021). Second, our findings demonstrate the differential predictive associations of LBLM: while constraining proactive innovation, LBLM simultaneously facilitates reactive innovation. This dual-effect pattern represents a significant departure from prior research emphasizing exclusively negative consequences.

Second, grounded in social information processing theory, this study examines the mechanism of willingness to take risks, uncovering its mediating role between LBLM and employee innovation behavior. This insight broadens the perspective on the mediating pathways that influence the effectiveness of LBLM. Social information processing theory posits that individual behaviors and motivations are often shaped by their interpretation of and response to social cues. Building on this theory, this study proposes that LBLM, as a mindset centered on risk avoidance and maintaining the status quo, significantly affects willingness to take risks by signaling a conservative organizational climate. However, in the context of reactive innovation, this risk-averse tendency may drive employees to adopt cautious innovation strategies under external pressures, ultimately fostering reactive innovation. These findings not only elucidate the mechanism linking LBLM to innovation behavior but also extend research on LBLM-mediated relationships. Prior studies have focused on the direct associations of LBLM (Hameed et al., 2025). By introducing willingness to take risks as a mediator, this study provides new theoretical support for how LBLM shapes employee behavior through psychological states.

Third, this study clarifies the boundary conditions of the effectiveness of LBLM by incorporating work values, thus opening new avenues for future research. Through empirical analysis, we demonstrate that work values moderate the relationship between LBLM and employee innovation behavior. Specifically, intrinsic work values weaken the negative predictive relationship between LBLM and willingness to take risks, whereas extrinsic work values exacerbate it, thereby indirectly shaping innovation outcomes. These findings advance the understanding of systematic associations of LBLM with employee behavior and underscore the pivotal boundary role of work values. Prior research has focused primarily on the direct relationships of LBLM (Mesdaghinia et al., 2019). By introducing work values as moderators, this study not only enriches the research framework of LBLM but also provides a basis for the future exploration of other contextual factors (e.g., organizational culture and leadership styles) as potential boundary conditions.

### 5.3 Practical implications

First, organizations should consider the following approaches to address the observed negative associations with LBLM: (1) Rigorous managerial screening: Avoid hiring candidates with dark triad traits (e.g., Machiavellianism, narcissism), as these individuals are more likely to adopt bottom-line thinking (Quade et al., 2020). (2) Comprehensive goal management training: Implement training programs to help managers balance financial objectives with other critical goals, such as social responsibility and employee well-being. (3) Open feedback channels: Establish transparent grievance mechanisms, enabling employees to report injustices promptly and effectively.

Second, leaders should foster employee willingness to take risks through proactive strategies: (1) Clarify strategic goals: Clearly communicate organizational objectives and role expectations, helping employees understand their responsibilities and significance within the broader mission. (2) Empower employees: Grant autonomy to encourage self-management and accountability, motivating employees to pursue innovative tasks. (3) Promote psychological safety: Incorporate flexibility and humanistic care into management practices, building trust and support to increase employee comfort with risk-taking.

Third, organizations should shift employees’ focus from extrinsic to intrinsic work values through the following measures: (1) Value-based hiring: Prioritize candidates with strong intrinsic work values (e.g., growth, purpose) over those with utilitarian orientations during recruitment. (2) Cultural alignment: Foster a corporate culture that emphasizes intrinsic values through education and dialogue, helping employees align their personal values with organizational goals. (3) Intervention training: Integrate work values training into employee development programs to promote employee internalization of intrinsic motivations, thereby encouraging innovation.

## 6 Limitations and directions

First, owing to research constraints, this study utilized cross-sectional data, which inherently limits the causal inference and cannot fully reveal the dynamic mechanisms among LBLM, willingness to take risks, work values, and employee innovation behavior (including both proactive and reactive innovation). Future research could employ longitudinal multiwave designs to better trace temporal dynamics and causal relationships. Experimental or quasi-experimental designs could further strengthen causal validity.

Second, while this study adopts a two-dimensional classification of work values (intrinsic and extrinsic), we acknowledge that classification may not fully capture their moderating roles. Research has suggested more nuanced dimensions, such as Super (2007) three-dimensional model (internal, extrinsic, and social rewards) or Schwartz (1999) four-dimensional framework (internal, extrinsic, social, and prestige). Future studies should examine how different value dimensions play distinct moderating roles, which would significantly advance work values research.

Third, while this study primarily examines willingness to take risks as mediator and work values as moderator, future research should explore additional mechanisms. Potential mediators may include psychological capital, emotional intelligence, and innovation self-efficacy, which could reveal alternative psychological pathways linking LBLM to innovation behavior. With respect to boundary conditions, multilevel contextual factors (e.g., organizational innovation climate, team cohesion) and interpersonal factors (e.g., perceived organizational support, psychological safety, LMX quality) warrant investigation. These factors may demonstrate differential moderating patterns in the observed predictive relationships, potentially mitigating the negative association of LBLM with proactive innovation while amplifying the benefits for reactive innovation.

Fourth, this study has limitations regarding sample gender distribution, with male employees constituting 66.38% of the participants, which may affect bias in the generalizability of findings across genders. Future research could benefit from (1) employing more balanced gender sampling strategies and (2) examining potential gender-based variations in the LBLM–innovation behavior relationships identified in this study. Such investigations would strengthen the external validity of these predictive findings.

Fifth, the measurement of willingness to take risks may exhibit cultural specificity. In Chinese organizational contexts, employees may systematically underreport their risk preferences in self-assessments due to the traditional mindset of “seeking neither merit nor fault.” While this study has mitigated bias through anonymous surveys and contextualized item design, future research is recommended to (1) employ multisource data (e.g., peer evaluation or behavioral experiments) for cross-validation; (2) develop localized scales suitable for high uncertainty avoidance cultures; and (3) incorporate reverse-scored items.

## Data Availability

The original contributions presented in the study are included in the article/supplementary material, further inquiries can be directed to the corresponding author.
